# Evaluating for unrecognized deficits in perimetry associated with functional upper eyelid malposition

**DOI:** 10.1016/j.aopr.2024.01.007

**Published:** 2024-02-02

**Authors:** Linyan Wang, Davin C. Ashraf, Michael Deiner, Oluwatobi O. Idowu, Seanna R. Grob, Bryan J. Winn, M Reza Vagefi, Robert C. Kersten

**Affiliations:** a Department of Ophthalmology, The Second Affiliated Hospital, Zhejiang University School of Medicine, Hangzhou, Zhejiang, China; b Department of Ophthalmology, University of California, San Francisco, USA; c Department of Ophthalmology, Oregon Health and Science University, Portland, USA; d Department of Ophthalmology, Tufts Medical Center, Boston, USA; e Department of Ophthalmology, University of Utah, Salt Lake City, Utah, USA

**Keywords:** Visual field, Ptosis, Dermatochalasis, Glaucoma, Perimetry, Visually significant

## Abstract

**Objective:**

To investigate whether functional upper eyelid malposition is associated with unrecognized deficits in automated perimetry among glaucoma patients by examining patients undergoing eyelid surgery who had not been identified as requiring eyelid taping during glaucoma field testing.

**Methods:**

In this retrospective pre-post study, an automated database search followed by manual chart review was used to identify eligible patients from January 2012 to March 2020. Included patients had reliable visual field testing within two years before and after functional upper blepharoplasty or ptosis repair and no comorbid ocular diagnoses. As part of routine practice, glaucoma visual field technicians taped patients with pupil-obstructing eyelid malposition; taped examinations were excluded from analysis. Clinical and demographic characteristics, mean deviation, and pattern standard deviation were evaluated within a two year period before and after eyelid surgery.

**Results:**

The final analysis included 60 eyes of 38 patients. Change in visual field parameters after eyelid surgery did not reach statistical significance in crude or adjusted analyses. Among patients with ptosis, the margin reflex distance-1 was not associated with change in mean deviation after surgery (Pearson R^2^ ​= ​0.0061; *P* ​= ​0.700). Five of 17 eyes excluded from analysis due to unreliable pre-operative visual fields demonstrated substantial improvement after surgery.

**Conclusions:**

Functional upper eyelid malposition does not appear to cause spurious visual field abnormalities among glaucoma patients with reliable visual fields who were determined not to require eyelid taping at the time of their visual fields. Unreliable visual fields could be a sign of eyelid interference in this population.

## 1 Introduction

Glaucoma is a leading cause of irreversible blindness worldwide, with both significant personal and societal burden.^1^ Visual field testing using automated perimetry is a crucial aspect of disease monitoring. A common visual field deficit in glaucoma involves an arcuate loss of superior sensitivity. Measures of visual field sensitivity are used to make diagnostic and therapeutic decisions, and are often used as the primary outcome in clinical trials of glaucoma treatment.^2^, ^3^, ^4^

Upper eyelid malposition, including blepharoptosis (ptosis) and dermatochalasis, may progress to occlude the superior, then central visual fields. When significant superior visual field loss is present, functional upper eyelid surgery may be carried out with robust evidence for visual field improvement.^5^ Severe upper eyelid malposition has been recognized as a potential cause of artifactual glaucomatous field loss.^6^^,^^7^ Due to the importance of visual field monitoring for diagnostic and therapeutic decisions, spurious progression of glaucoma due to upper eyelid malposition is of concern. Eyelid taping is often carried out to improve apparent eyelid abnormalities during glaucoma field testing. In experimental settings, patients with overt eyelid abnormalities have demonstrated improvement in visual field parameters upon taping.^8^ Notably, eyelid taping requires recognition of visually significant ptosis/dermatochalasis, and thus some cases may not be appropriately taped. Furthermore, visual field deficits that improved only with surgery and not with taping have been reported.^6^ Therefore, the authors sought to investigate the effect of eyelid surgery on visual field parameters among glaucoma patients, in order to evaluate whether eyelid malposition might have produced spurious visual field loss.

## 2 Methods

This retrospective study was conducted at the Department of Ophthalmology, University of California, San Francisco (UCSF), USA, with approval from the Institutional Review Board (IRB) (#19–29672). This study adhered to the tenets of the Declaration of Helsinki as amended in 2013 and was compliant with the Health Insurance Portability and Accountability Act. The requirement for informed consent was waived by the IRB due to the retrospective and minimal-risk nature of the study.

### 2.1 Data extraction

Current Procedural Terminology (CPT) codes were used for initial patients’ data screening. The cohort was generated from outpatient visits occurring between January 2012 and March 2020 with billing for both the automated perimetry CPT code 92083 (Humphrey visual field 10–2, 24–2, 30–2, 60–2 or equivalent) and any of the upper eyelid surgery CPT codes, including 67903 (internal levator resection), 67904 (external levator resection), 15822/15823 (upper blepharoplasty). Manual chart review was then performed to determine eligibility for the study. Included patients had: a diagnosis of glaucoma by an ophthalmologist or optometrist; an operative report available documenting functional upper eyelid surgery to correct dermatochalasis or ptosis; and reliable automated perimetry for glaucoma monitoring within two years before and after the eyelid surgery. Visual field reliability was determined based on previously reported criteria^9^: fixation loss <20%; false positive errors <15%; false negative errors <15%. Exclusion criteria included: documentation of eyelid taping during the visual field; diagnosis of glaucoma suspect; cosmetic upper eyelid surgery; post-operative documentation of persistent visually significant dermatochalasis or margin reflex distance-1 (MRD-1) of <3 ​mm; history of vision loss due to other diseases associated with optic nerve or retinal dysfunction, such as vascular occlusive disease, uveitis, or diabetic retinopathy.

The cosmetic or functional nature of upper eyelid surgery was based on surgeon documentation. In the United States, functional upper blepharoplasty or ptosis repair is carried out when the superior visual field is obstructed by the eyelids, and the resulting deficit improves with manual elevation of the lids. The specific criteria for demonstrating superior visual field obstruction varies by insurer; Medicare, a common public insurance plan among the demographic in this study population, requires a minimum loss of 12° or 30% of the superior field attributable to the malposition, with demonstration of improvement upon manual elevation.^10^

The following data were abstracted from the medical record: demographic information; history of glaucoma treatments including drops and surgeries; major perimetric parameters of visual fields (mean deviation, MD; pattern standard deviation, PSD). The MD is the average depression (or elevation) of the measured visual field compared to age-matched controls.^11^ Pattern standard deviation (PSD) measures irregularity by summing the absolute value of the difference between the threshold value for each point and the average visual field sensitivity at each point (equal to the sum of the age-normal value for each point and the MD).^12^ The MD and PSD were chosen as the primary measures due to their objective, quantitative nature.

### 2.2 Equipment and clinical practice

Patients completed automated perimetry on the Humphrey Field Analyzer 750i and 850i (Carl Zeiss AG, Germany) under supervision of certified ophthalmic technicians. Technicians at the authors' institution are trained to tape the upper eyelid when noting any degree of obscuration of the undilated pupil by a ptotic eyelid or excess upper eyelid skin. Technicians document the taping in the electronic medical record. To determine eligibility for functional upper eyelid surgery, the authors’ institution utilizes the Humphrey Superior 36 Point pattern, which assesses the superior 60-degree hemifield.^13^

### 2.3 Statistical analysis

Data analysis was performed using R (R Core Team, 2020).^14^ Crude analysis utilized the paired *t*-test to assess change in MD and PSD before and after eyelid surgery. Multiple linear regression was utilized to adjust for potential confounders including age at the time of each visual field, gender, type of eyelid surgery, and ethnicity. A mixed effects model was chosen with random effects to account for intrasubject correlation between the two eyes. A *P*-value of <0.05 was considered significant. Graphical charts were generated using the package gg-plot.^15^

## 3 Results

A total of 489 patients had CPT codes consistent with glaucomatous visual field tests between January 2012 and March 2020 and were included for initial screening. Among these 489 patients, 145 patients had CPT codes for relevant upper eyelid surgeries. The CPT codes 15822, 15823, 67903, and 67904 were associated with 18, 59, 41, and 37 patients, respectively (with some patients undergoing more than one surgery on the same day). Manual review of the date associated with each CPT code identified 103 patients for whom visual fields were coded within 2 years before and after surgery. Finally, chart review found 60 eyes of 38 patients who met eligibility criteria for inclusion in the final analysis. Demographic and clinical characteristics are summarized in Table 1.

**Table 1:** Clinical and demographic characteristics.

	Mean (SD) or N (%)
**Age (years)**	72.7 (10.5)
**Gender**	
Female	25 (65.8%)
**Ethnicity (patients)**	
Asian	18 (47.4%)
White	12 (31.6%)
Hispanic	7 (18.4%)
Black	1 (2.6%)
**Type of Surgery (eyes)**	
Ptosis repair	36 (60%)
Pre-operative MRD-1 (mm)	0.53 (0.66)
vBlepharoplasty	28 (46.7%)
**Visual Field Timing (months)**	
Before Surgery	9.4 (7.4)
After Surgery	12.5 (14.9)
**Field Type (eyes)**	
10-2	4 (6.7%)
24-2	35 (58.3%)
30-2	21 (35%)

MRD-1 ​= ​Margin reflex distance-1; SD = Standard deviation.

### 3.1 Change in visual field parameters and glaucoma regimen after surgery

**Fig. 1:**
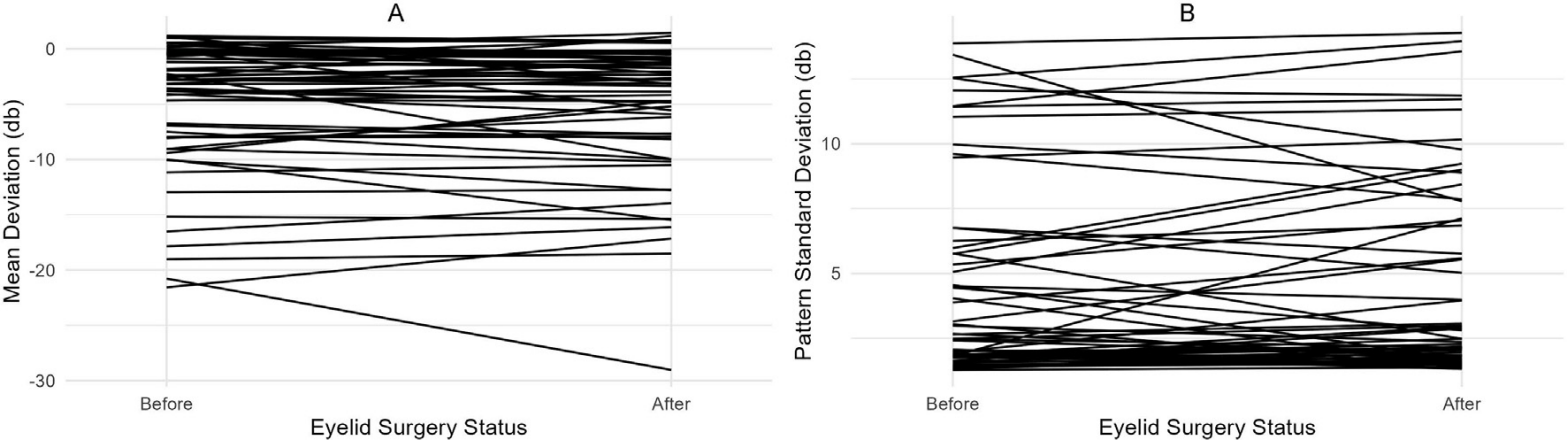
Change in Visual Field Parameters Before and After Eyelid Surgery (A) Change in mean deviation. (B) Change in pattern standard deviation.

Average MD and PSD were not significantly different before and after eyelid surgery (Table 2); four eyes (6.7%) had ≥3 ​dB improvement in MD. Data visualization demonstrated marked variation for individual patients accounting for minimal aggregate change (Fig. 1). Similarly, eyelid surgery was not found to be significantly associated with MD or PSD in the adjusted analysis with control for potential confounders (Table 2). The variance inflation factors for age, gender, ethnicity, and eyelid surgery were low (Supplemental Table 1).

**Table 2:** Comparison of pre-operative and post-operative visual field parameters.

	Pre-Surgery Mean (SD)	Post-Surgery Mean (SD)	Mean Difference (SD)	*P*-value^a^	Mean Difference, Surgery Coefficient (SE) [95% CI]	Mean Difference, Time Coefficient, per Year (SE) [95% CI]	*P*-value^b^
**Mean Deviation (dB)**	−4.80 (5.81)	−5.16 (6.04)	−0.36 (1.08)	0.249	−0.32 (0.59) [−1.48, 0.85]	−0.04 (0.09) [−0.21, 0.14]	0.590
**Pattern Standard Deviation (dB)**	4.44 (3.77)	4.53 (3.79)	0.10 (0.69)	0.666	0.04 (0.36) [−0.67, 0.76]	0.05 (0.06) [−0.06, 0.16]	0.520

CI = Confidence interval; dB ​= ​decibel; SD = Standard deviation; SE = Standard error.

a Crude analysis performed with paired *t*-test.

b Adjusted analysis performed with a mixed effects linear regression model using random effects to account for intrasubject correlation between the two eyes, and including age at the time of each visual field, gender, and ethnicity as predictors to account for potential confounding. Regression coefficients for eyelid surgery and age at the time of each visual field were used to estimate relative contributions to the mean difference.

The relationship between predictor variables and change in MD and PSD was also explored (Table 3). Type of eyelid surgery was not associated with change. Among patients with ptosis, pre-operative MRD-1 was not associated with change in MD after surgery (Pearson R^2^ ​= ​0.0061; *P* ​= ​0.700). Only female gender was found to be significantly associated with change in MD between the pre- and post-eyelid surgery visual fields. No difference was noted in the mean number of glaucoma medications before and after surgery (0.97 ​± ​0.90 vs. 1.06 ​± ​0.91; *P* ​= ​0.263).

**Table 3:** Associations between change in visual field parameters and potential.

	Change in Mean Deviation (dB)		Change in Pattern Standard Deviation (dB)	
	Mean (SE) [95% CI]	*P*-value^a^	Mean (SE) [95% CI]	*P*-value^a^
Eyelid surgery				
No surgery	reference		reference	
Ptosis repair	−0.17 (1.38) [−2.72, 2.40]	0.901	0.01 (0.92) [−1.72, 1.74]	0.990
Blepharoplasty	−1.70 (1.41) [−4.30, 0.95]	0.236	1.37 (0.94) [−0.41, 3.15]	0.150
Time (per year)	0.28 (0.25) [−0.19, 0.75]	0.277	−0.20 (0.16) [−0.51, 0.10]	0.216
Gender (Female)	1.36 (0.70) [0.08, 2.65]	0.059	−0.91 (0.45) [−1.76, −0.06]	0.049
Ethnicity (Asian)	1.16 (0.70) [−0.17, 2.46]	0.110	−0.36 (0.46) [−1.24, 0.51]	0.437

CI = Confidence interval; dB ​= ​decibel; SD = Standard deviation; SE = Standard error.

a Mixed effects linear regression model using random effects to account for intrasubject correlation between the two eyes, with change in mean deviation or pattern standard deviation as the dependent variable.

### 3.2 Excluded cases

**Fig. 2:**
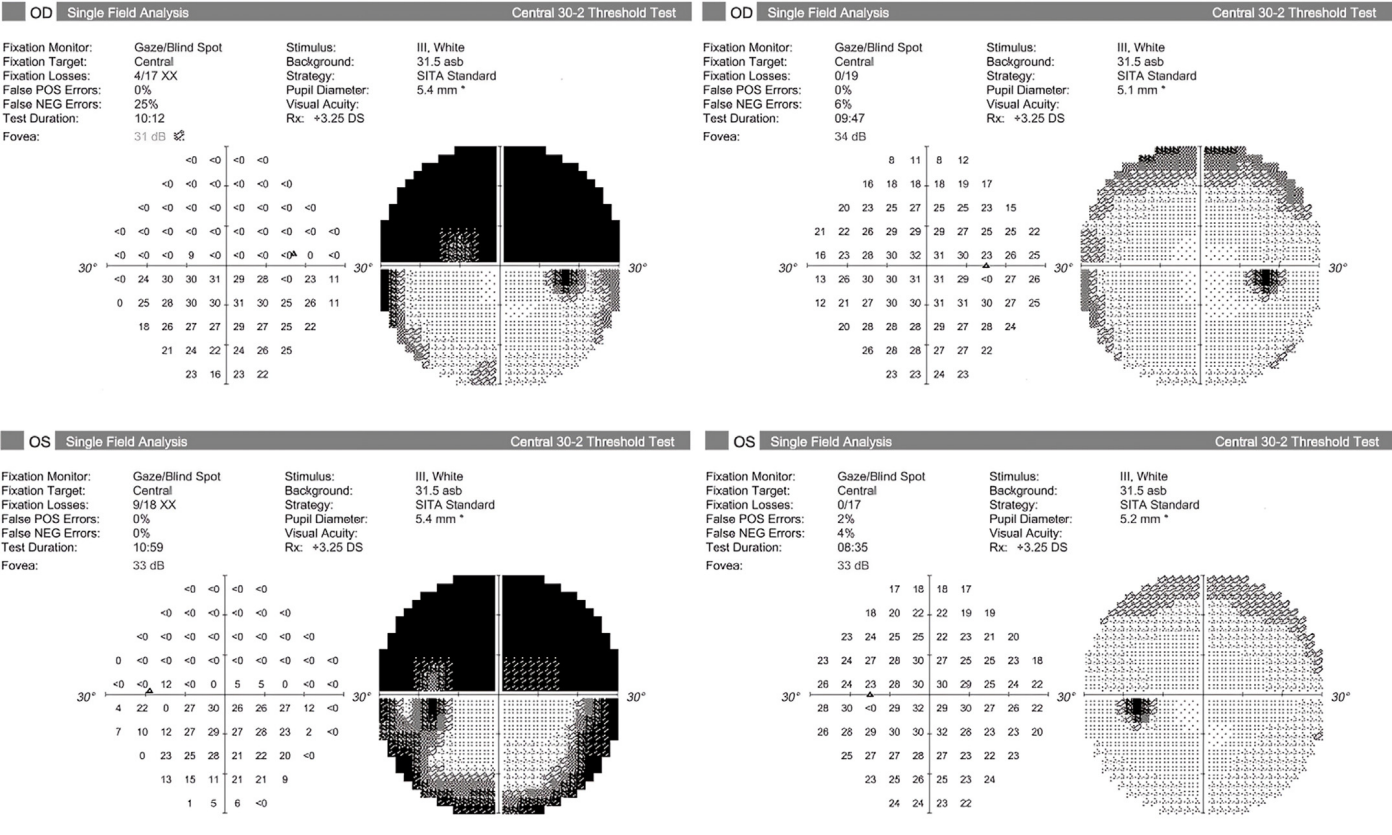
Peri-operative Visual Field Testing in an Excluded Patient. The left panel demonstrates pre-operative visual field testing for an excluded patient. These fields were ineligible for inclusion due to high fixation loss and false negative errors. There is a dense superior hemifield defect that largely normalizes after eyelid surgery on the post-operative fields shown in the right panel.

Manual chart review excluded 65 patients for failing to meet study criteria. Among these, 10 patients had 17 eyes that were excluded solely because of unreliable glaucoma visual fields in the two-year pre-operative period. Seven of these eyes (41.2%) had ≥3 ​dB improvement in MD, and five (29.4%) had marked superior visual field defects evident on manual review that improved with eyelid surgery. An example is shown in Fig. 2. In this subpopulation of excluded cases, MD improved after eyelid surgery (pre-operative −7.72 ​± ​6.27 ​dB vs. post-operative −3.54 ​± ​3.68 ​dB; *P* ​= ​0.003). As a sensitivity analysis to determine whether altering the inclusion criteria would have affected the results of the primary analysis, a paired *t*-test was performed on the study population with the addition of these 17 excluded eyes. No significant difference in MD between time points was noted (pre-operative −5.55 ​± ​6.14 ​dB vs. post-operative −4.93 ​± ​5.69 ​dB; *P* ​= ​0.148).

## 4 Discussion

In this study, the authors retrospectively compared reliable visual field parameters of glaucoma patients collected before and after the correction of visually significant dermatochalasis or blepharoptosis. Using PubMed search terms ("blepharoplasty"; "ptosis repair"; "internal levator resection"; "external levator resection"; "glaucoma"; "visual field"; "automated perimetry"), the authors were unable to find a comparable study population. Kosmin et al. had evaluated a series of patients with dermatochalasis and healthy optic nerves but superior visual field deficits concerning for glaucoma, and had found an improvement in MD from −6.66 to −2.46 ​dB with lid taping or surgery.^7^ Similarly, Nesher et al. identified patients with severe ptosis and superior hemifield defects and found an improvement in MD from −8.85 to −6.92.^8^ However, as these populations were specifically chosen based on disease severity or incongruity, they are not comparable. The present study is unique in including an institution's general population of glaucoma patients with functional upper eyelid malposition to evaluate for spurious glaucomatous field changes.

The primary finding in this series was a lack of change in MD or PSD after eyelid surgery, with 95% confidence intervals for change clustered around zero. Notably, the study population excluded cases with unreliable visual fields, as well as cases for which the glaucoma team noted apparent pupillary obstruction and taped the eyelids at the time of visual field examination. Therefore, this study's findings suggest that glaucoma patients with reliable, untaped visual fields at an institution with protocolized taping are not likely to suffer unrecognized visual field deficits as a result of upper eyelid malposition. Correspondingly, no difference was identified in the number of glaucoma medications before and after surgery, suggesting that management did not change.

Experimental data provides some context for this study's counterintuitive findings. Meyer et al.^16^ used partially opaque contact lenses to simulate obstruction of the visual axis by the upper eyelid at different levels of pseudo-MRD-1. Using this technique, they correlated an MRD-1 of 0.5–1.0 ​mm to the superior 30-degree field and −0.5 to 0 ​mm to the superior 20-degree field. Similarly, Goldman perimetry has been used to demonstrate that 97% of patients with an MRD-1 of less than 2 ​mm have constriction of the superior visual field to 30° or less.^16^ The mean pre-operative MRD-1 among patients with ptosis in the current study population was 0.53 ​mm, suggesting that some occlusion might be expected on the popular 24–2 and 30–2 protocols. The lack of an observed difference in the present study may be due to limited power to detect a small superior visual field defect that improves with surgery; however, the narrow 95% confidence interval of change in field parameters argues against this. More likely, the authors suspect that patient compensation for eyelid malposition explains the incongruity in this study population.

Measurement of MRD-1 for ptosis evaluation (or pseudo-MRD-1 for dermatochalasis) involves neutralization of frontalis muscle activity to demonstrate the full extent of the eyelid pathology; on the contrary, frontalis recruitment to clear the visual axis is desirable and reflexive at other times, including during examination and visual field testing in the glaucoma clinic. By excluding patients who appeared to require taping, this study likely selected for patients with robust compensation for an upper eyelid malposition that had been determined to be functional (i.e. MRD1 ≤2 ​mm with superior field loss) when the frontalis muscle was intentionally relaxed on oculoplastic evaluation.

Interestingly, patients who were excluded due to unreliable pre-operative visual fields demonstrated a higher rate of improvement by ​≥ ​3 ​dB in MD than the study population (41.2% vs 6.7%; *P* ​< ​0.001, Chi-squared test). Furthermore, a small group was found on manual review to have dense, complete or superior loss on pre-operative testing that improved on post-operative testing, apparently due to eyelid malposition similar to the case reported by Provencher et al.^6^ Presumably, this represents a subset of patients who are less able to compensate for their eyelid pathology. Taken together, a reasonable conclusion may be that a taping protocol offers reassurance against spurious visual field anomalies on glaucoma perimetry, except in the event of unreliable visual fields, which should raise the index of suspicion for interference by eyelid pathology.

This study has several limitations. The design was retrospective and therefore clinical measurements and testing were performed without a consistent schedule in relationship to surgery. To account for this, visual fields were evaluated within 2 years of surgery as most glaucoma patients will receive periodic testing within this time frame. Unfortunately, this window does raise the possibility of progression due to glaucoma or normal aging during the interval between fields. Prior research estimates that normal aging leads to an average change in MD of −0.06 ​dB/year, and glaucomatous deterioration between −0.05 and −0.62 ​dB/year.^17^ Thus, using the mean time between fields of 22 months and the high end of these estimates, the error induced might approach −1.13 ​dB in this study, which exceeds the observed change of −0.36 ​dB and potentially masks a small amount of improvement related to eyelid surgery. Notably, the difference of 0.77 ​dB would generally not be considered a clinically significant change in visual field requiring escalation of glaucoma therapy. While clinical protocol dictates that the visual field technician should document eyelid taping when performed, the lack of a prospective study protocol could have led to inadvertent inclusion of taped cases that were not documented as such. The inclusion criteria may have introduced selection bias. The exclusion of eyes that were taped during visual field testing selects against the most severe pathology and precludes evaluation of taping efficacy (i.e. identification of spurious deficits among taped patients); however, there is little reason to doubt that severe eyelid malpositions affect perimetry for glaucoma, and the efficacy of taping has been previously evaluated.^8^ Unreliable visual fields inherently have the potential to introduce noise into the dataset and thus were excluded during study design. This may have reduced the observed effect of surgery based on our post-hoc evaluation of the excluded population, but sensitivity analysis involving inclusion of these fields did not significantly alter the results. Finally, MD and PSD are fairly global measures of the visual field, and may be insensitive to subtle superior visual field loss; however, they can be objectively interpreted and can be used to determine progression across glaucoma stages.^18^, ^19^, ^20^ The authors elected to avoid subjective grading of the fields, which may have had an advantage in evaluating subtle superior loss, in favor of an objective method.

## 5 Conclusions

In conclusion, a glaucoma practice that routinely tapes pupil-obstructing upper eyelid malposition can be reassured that those patients with upper eyelid malposition not meeting taping criteria are unlikely to harbor unrecognized visual field changes owing to their eyelid disease, so long as their visual fields remain reliable. An unreliable test could be a sign of eyelid interference and any upper eyelid malposition should be evaluated with suspicion in such a case.

## Study approval

This retrospective study was conducted at the Department of Ophthalmology, University of California, San Francisco (UCSF), USA, with approval from the Institutional Review Board (IRB) (#19–29672). This study adhered to the tenets of the Declaration of Helsinki as amended in 2013 and was compliant with the Health Insurance Portability and Accountability Act. The requirement for informed consent was waived by the IRB due to the retrospective and minimal-risk nature of the study.

## Author contributions

The authors confirm contribution to the paper as follows: Conception and design of study: DA, LW; Data collection: DA, LW; Analysis and interpretation of results: DA, LW; Drafting the manuscript: all authors; All authors reviewed the results and approved the final version of the manuscript.

## Funding

Supported in part by NIH-10.13039/100000053NEI P30 EY002162–Core Grant for Vision Research and by an unrestricted grant from 10.13039/100001818Research to Prevent Blindness.

## Declaration of competing interest

Dr. Oluwatobi O. Idowu became employed by AbbVie Inc. as a Medical Science Liaison during the period of manuscript preparation. The other authors have no disclosures that might influence this work.

## Abbreviations

CPT: Current procedural terminology
ICD: International classification of disease
MD: Mean deviation
MRD-1: Margin reflex distance-1
PSD: Pattern standard deviation
