# Supplementary material for: Evaluating for unrecognized deficits in perimetry associated with functional upper eyelid malposition
Source: Adv Ophthalmol Pract Res. 2024 Feb 2;4(1):39–44. doi: 10.1016/j.aopr.2024.01.007 (PMC10891280; doi:10.1016/j.aopr.2024.01.007)
Supplement: Multimedia component 1 [file mmc1.docx]

**Supplementary data**

**Supplemental Table 1. Variance Inflation Factors**

| Lid Surgery | 1.00 |
| --- | --- |
| Age | 1.03 |
| Female Gender | 1.02 |
| Asian Ethnicity | 1.02 |
